# Efficacy of a biomechanically-based yoga exercise program in knee osteoarthritis: A randomized controlled trial

**DOI:** 10.1371/journal.pone.0195653

**Published:** 2018-04-17

**Authors:** Alexander B. Kuntz, Jaclyn N. Chopp-Hurley, Elora C. Brenneman, Sarah Karampatos, Emily G. Wiebenga, Jonathan D. Adachi, Michael D. Noseworthy, Monica R. Maly

**Affiliations:** 1 Department of Kinesiology, McMaster University, Hamilton, Ontario, Canada; 2 School of Rehabilitation Sciences, McMaster University, Hamilton, Ontario, Canada; 3 Department of Medicine, McMaster University, Hamilton, Ontario, Canada; 4 Department of Electrical and Computer Engineering, McMaster University, Hamilton, Ontario, Canada; 5 Department of Kinesiology, University of Waterloo, Waterloo, Ontario, Canada; IRCCS E. Medea, ITALY

## Abstract

**Objective:**

Certain exercises could overload the osteoarthritic knee. We developed an exercise program from yoga postures with a minimal knee adduction moment for knee osteoarthritis. The purpose was to compare the effectiveness of this biomechanically-based yoga exercise (YE), with traditional exercise (TE), and a no-exercise attention-equivalent control (NE) for improving pain, self-reported physical function and mobility performance in women with knee osteoarthritis.

**Design:**

Single-blind, three-arm randomized controlled trial.

**Setting:**

Community in Southwestern Ontario, Canada.

**Participants:**

A convenience sample of 31 women with symptomatic knee osteoarthritis was recruited through rheumatology, orthopaedic and physiotherapy clinics, newspapers and word-of-mouth.

**Interventions:**

Participants were stratified by disease severity and randomly allocated to one of three 12-week, supervised interventions. YE included biomechanically-based yoga exercises; TE included traditional leg strengthening on machines; and NE included meditation with no exercise. Participants were asked to attend three 1-hour group classes/sessions each week.

**Measurements:**

Primary outcomes were pain, self-reported physical function and mobility performance. Secondary outcomes were knee strength, depression, and health-related quality of life. All were assessed by a blinded assessor at baseline and immediately following the intervention.

**Results:**

The YE group demonstrated greater improvements in KOOS pain (mean difference of 22.9 [95% CI, 6.9 to 38.8; p = 0.003]), intermittent pain (mean difference of -19.6 [95% CI, -34.8 to -4.4; p = 0.009]) and self-reported physical function (mean difference of 17.2 [95% CI, 5.2 to 29.2; p = 0.003]) compared to NE. Improvements in these outcomes were similar between YE and TE. However, TE demonstrated a greater improvement in knee flexor strength compared to YE (mean difference of 0.1 [95% CI, 0.1 to 0.2]. Improvements from baseline to follow-up were present in quality of life score for YE and knee flexor strength for TE, while both also demonstrated improvements in mobility. No improvement in any outcome was present in NE.

**Conclusions:**

The biomechanically-based yoga exercise program produced clinically meaningful improvements in pain, self-reported physical function and mobility in women with clinical knee OA compared to no exercise. While not statistically significant, improvements in these outcomes were larger than those elicited from the traditional exercise-based program. Though this may suggest that the yoga program may be more efficacious for knee OA, future research studying a larger sample is required.

**Trial registration:**

ClinicalTrials.gov (NCT02370667)

## Introduction

Knee osteoarthritis (OA) is associated with pain, mobility limitations, and a variety of comorbidities such as cardiovascular, gastrointestinal, and metabolic diseases, as well as depression [1]. Conservative treatment options are necessary to make meaningful use of time spent using analgesics and, for some, the multi-year gap between diagnosis and joint replacement. Exercise provides equivalent pain relief to medication while also improving physical function, comorbidities, and quality of life [2–4]. However, some features of common exercises can exacerbate symptoms and contribute to disease progression. For example, exposure to elevated magnitudes of the knee adduction moment (KAM), a mechanical variable reflecting the ratio of medial to total knee loading, predicts disease progression [5–8], with repetitive exposure linked to pain severity in OA [9]. Therefore, it is critical to ensure that exercise prescriptions for knee OA minimize exposure to KAM.

Yoga may be an ideal exercise option for knee OA. If the correct postures are selected, exposure to end-range joint positions and large KAM can be reduced [10,11]. Yoga also involves cultivating “mindfulness”; that is, paying deliberate attention in a non-judgmental manner to one’s experience of the present moment [12]. Mindfulness practice can ameliorate pain in chronic conditions like arthritis [13,14]. This is an important paradigm for interventions aimed to address the symptoms of OA that are both physiological and psychological [15,16]. Although a lack of high quality evidence exists, existing literature suggests yoga is a promising and safe treatment for OA [17,18]. In a 12-week cohort study of women with knee OA, we previously demonstrated that yoga reduced pain, increased knee muscle strength, and improved physical function and mobility performance [10]. It remains unclear how the improvements in symptoms, mobility and physical capacity compare between this novel yoga exercise program based on biomechanical principles [11] versus a traditional exercise program prescribed for OA.

The purpose of this study was to compare the effectiveness of a biomechanically-based yoga exercise intervention to traditional exercises for knee OA, and an attention-equivalent no exercise control group in women with clinical knee OA. The primary outcomes were pain, self-reported physical function and mobility performance. We also investigated knee muscle strength, depression, and health-related quality of life. It was hypothesized that the yoga group would experience greater improvements in all outcomes compared to the no exercise group; and equal or greater improvements compared to traditional exercise group.

## Materials and methods

### Design overview

This study was a single-blind, three-arm, parallel, randomized controlled trial.

### Setting and participants

This trial (NCT02370667) was conducted at McMaster University in Hamilton, ON, Canada and was approved by the Hamilton Integrated Research Ethics Board (#15–021) where all participants provided written informed consent.

A sample size calculation for an analysis of covariance (ANCOVA) designed to detect significant differences in three primary outcomes between three groups with one covariate (baseline values) was performed. A systematic review of yoga as a therapeutic intervention for adults with chronic pain concluded yoga is capable of reducing pain by a standard mean difference of -0.74 (95% CI, -0.97 to -0.52; P < 0.0001) [19]. Given this moderate effect size, high correlation between outcomes, a 5% chance of type one error (two-sided) and 15% attrition, a sample of 60 participants was recommended to yield 80% power to detect between group differences.

Participants were recruited through rheumatology, orthopaedic, and physical therapy clinics, as well as by word-of-mouth and newspaper advertisements in the Hamilton, Ontario, Canada region between April and June 2015. The sample included ambulatory, community-dwelling women, 50 years of age or over, who met the diagnostic criteria for clinical knee OA according to the American College of Rheumatology [20]. Clinical OA diagnostic criteria were chosen given that radiographic disease severity does not appear to influence the effectiveness of exercise [21]. Exclusion criteria consisted of other forms of arthritis, history of osteoporotic fracture, patellofemoral pain, non-arthritic knee disease, knee surgery, unstable heart condition, neurological conditions, physician-advised physical activity restrictions, skin allergy to medical tape, lower limb trauma in past three months, ipsilateral hip or ankle conditions, undergoing cancer treatment, and pregnancy.

### Randomization and interventions

Participants were randomized to one of the three study interventions after stratification for disease severity. Disease severity was determined using the Lower Extremity Functional Scale (LEFS), [22,23] a 20-item measure addressing lower extremity physical function limitations associated with musculoskeletal conditions affecting the lower extremity. This tool is scored out of 80, where higher scores indicate better function. It was designed such that it is easy to administer and score and applicable to a wide range of people with lower limb conditions. Mild limitation was regarded as LEFS scores between 51 and 65, and those between 30 and 50 were moderate [24]. After stratification, randomization was performed using custom Matlab® software, with a block size of n = 6 and 1:1:1 allocation ratio. The intervention arms included a yoga exercise (YE) experimental group, a traditional exercise (TE) active treatment comparison group, and a no exercise (NE) attention-equivalent control group. Participants received group allocation information in an opaque envelope. This process was completed by an investigator who was not involved in data collection. All data collection was led by an investigator who was blind to group allocation and uninvolved in the interventions. Participants and exercise instructors were blinded to the study hypothesis. All three interventions were 12-weeks in duration. For each of the three interventions, participants were asked to attend three of four available one-hour classes/sessions each week. The classes/sessions were supervised. The interventions took place between June and September 2015 in Hamilton, Ontario, Canada.

The group YE intervention was led by a certified, trained yoga instructor. YE consisted of alignment-based postures that activate the lower limb musculature while maintaining a low KAM [10,11]. The selected weight-bearing, static poses were performed barefoot and included squats and lunges with varying foot, trunk, and arm positioning. Careful attention was given to ideal alignment of the leg throughout the exercises. The classes began with a body-awareness exercise performed in supine followed by the strengthening postures and concluded with a closing deliberate relaxation exercise performed in supine. Exercise difficulty was progressively increased over the 12-week intervention period.

The TE intervention reflected the current gold standard of strengthening exercise for knee OA [2]. The program emphasized knee strengthening but also involved an aerobic warm-up, balance exercises, and stretching. TE was designed and supervised by kinesiologists and physical therapists and took place at a physical activity center. The sessions involved a ten-minute warm-up performed on a treadmill or cycle ergometer. Then, lower extremity strengthening was performed on pneumatically-resisted exercise machines (HUR USA, Inc., Northbrook, IL, USA). Exercises included all major muscle groups of the lower extremity. The quadriceps were targeted at every session. Participants also completed balancing activities and static stretching. There was a progressive increase in the number of sets and resistance during strengthening exercises over the course of the intervention.

In both YE and TE, participants were asked to exercise at an intensity of seven out of ten on the Borg Perceived Exertion Scale [25]. Participants were also asked to rate their knee pain on a visual analog scale [26] prior to each session and ensure that pain levels were not exacerbated by more than two points. In the event that pain increased more than two points during a session, participants were asked to notify the instructor for an appropriate modification [10].

The NE intervention consisted of group-based, guided meditative relaxation classes led by a certified yoga-instructor. These sessions included non-physically active somatic awareness exercises including breath and body-scan meditation practices performed in passive postures fully supported by the use of yoga props.

Strategies to enhance participant adherence included a gift bag after the initial data collection visit, rewards for best attendance halfway through intervention, and a $50 stipend upon study completion. Session attendance and program adherence was monitored.

### Outcomes and follow-up

All outcomes were measured before and immediately after the 12-week intervention and led by the same blinded assessor. The primary outcomes were pain, self-reported physical function and mobility performance. Multiple primary outcomes were selected to ensure that both self-reported and performance-based measurements were included, since these outcomes reflect unique elements of OA disease [27–29]. Further, mobility performance was assessed using the set of tools that is recommended for interventional studies of knee OA [30]. Secondary outcomes included muscle strength, symptoms of depression, and health-related quality of life. Because OA affects multiple elements of health [31]; and the mechanisms by which yoga improves health appears multifactorial [19], these broader constructs relevant to health and well-being were explored. Adverse events were tracked by having participants complete a report at follow-up that asked whether they experienced an event that may have affected their quality of life or function since the last visit. This report was standard across participants.

#### Primary outcomes

Pain was assessed using the pain subscale of the Knee Injury and Osteoarthritis Outcome Score (KOOS) and the Measure of Intermittent and Constant Osteoarthritis Pain (ICOAP). The KOOS pain subscale yields a score of pain intensity during a variety of movements and activities. It is a nine-item tool answered on a five-point Likert scale. Scores are normalized out of 100; lower scores indicate more extreme and troublesome symptoms. In contrast to the KOOS, the ICOAP provides information on pain intensity and frequency, and the consequent effects on aspects of life independent of the effects of pain on physical function. The ICOAP is an 11-item questionnaire where higher scores are indicative of more severe pain. The tool was designed to distinguish the OA pain experience into constant pain and intermittent pain, and yields scores for the most troublesome joint and the resulting impact on mood, sleep, and quality of life. Both the KOOS and ICOAP produce valid and reliable data in adults with knee OA [32,33].

Self-reported physical function was assessed using the LEFS, as well as the function in activities of daily living (ADL) and sport and recreation (SR) subscales of the KOOS. The LEFS has 20 items that assess difficulty during mobility tasks. This tool avoids ceiling effects in high functioning samples and has superior sensitivity and discriminant validity than KOOS subscales [34]. Low LEFS scores represent poor mobility [22]. The ADL and SR subscales are 17-item and 5-item tools respectively; where higher scores indicate better function.

Mobility performance measures included those recommended by the Osteoarthritis Research Society International: the six-minute walk (SMWT), 40-meter walk (40mW), 30-second chair stand (30sCS), timed up and go (TUG), and stair ascent (SA) tests [35]. In the SMWT test, participants were instructed to walk as far as possible in six-minutes; the distance travelled was recorded. Time spent walking the initial 40 meters of the SMWT was used as 40mW score. The number of times participants were able to rise from and return to a chair in 30 seconds was the 30sCS score. The TUG test involved measuring the duration of time required for participants to rise from a chair, walk three meters, and return to their seat. All of these mobility performance measures produce valid and reliable data in individuals with knee OA [35–38]. Lastly, time taken to ascend a 9-step staircase as quickly as possible, with or without the use of a handrail, was recorded for the SA test. This stair climbing assessment produced reliable data (ICC 0.72–0.88, SEM <0.4s) in 29 healthy adults in our laboratory.

#### Secondary outcomes

Muscle strength was represented by peak torque of the knee extensor and flexor muscle groups of participants’ most symptomatic knee during maximal voluntary efforts. Participants were positioned on a dynamometer (Biodex System 2, Biodex Medical Systems, Shirley, NY, USA) with the knee joint center aligned with the device axis of rotation and in 65° of flexion relative to full extension. Torso, pelvis, thigh, and lower leg restraints were used to minimize the contribution of other muscle groups. Apparatus settings were recorded for each participant at baseline and replicated during follow-up for consistency. Participants completed five maximal voluntary isometric muscle actions following a submaximal, isotonic warm-up and familiarization. Each effort lasted five seconds, with five seconds of rest between bouts. Participants were provided with verbal encouragement and visual feedback to maximize voluntary effort. The peak torque value obtained during these five efforts was expressed relative to body mass (Nm/kg).

Symptoms of depression were evaluated using the Center for Epidemiological Studies Depression Scale (CESD). The CESD is a 20-item tool inquiring about affect (mood, guilt, worthlessness, helplessness, appetite, and sleep) that produces valid and reliable data in the general population and individuals with rheumatoid arthritis [39,40]. Quality of life was assessed using the four-item knee related quality of life (QoL) subscale of the KOOS.

### Statistical analyses

Descriptive statistics were calculated. A one-way ANOVA was used to detect whether differences in age, BMI and LEFS score existed between groups baseline. An ANCOVA comparing mean change scores across the intervention (follow-up minus baseline), with baseline data used as covariates in the models were used to detect between-group differences for each outcome. Sidak adjustments were performed to account for multiple comparisons between groups; alpha values of 0.05 were used. Assumptions of ANCOVA were tested and met [41]. Data distribution was assessed visually and using Shapiro-Wilk tests; homoscedasticity was assessed using Levene’s test for equality of error variance; independence of covariate and treatment effect was assessed using a one-way analysis of variance (ANOVA); and homogeneity of regression slopes of covariates versus dependent variables was assessed visually and by testing group allocation by baseline score interactions in an ANOVA. Paired (two-tailed) t-tests between pre and post intervention group means were also calculated to detect within-group differences. A Bonferroni correction was used to adjust for multiple within-group comparisons; an alpha value of 0.0167 was used. Statistical analyses were conducted using SPSS v. 23 (IBM, IL, USA). Additionally, to evaluate clinical significance, outcomes were interpreted relative to the minimal clinically important differences (MCID) and patient acceptable symptoms states (PASS). The MCID represents the smallest increment of change that a patient would identify as important. The PASS values are those in which scores equal or above are deemed “unacceptable” to live with by individuals with OA [42].

## Results

### Participant recruitment, retention, and adherence

**Fig 1:**
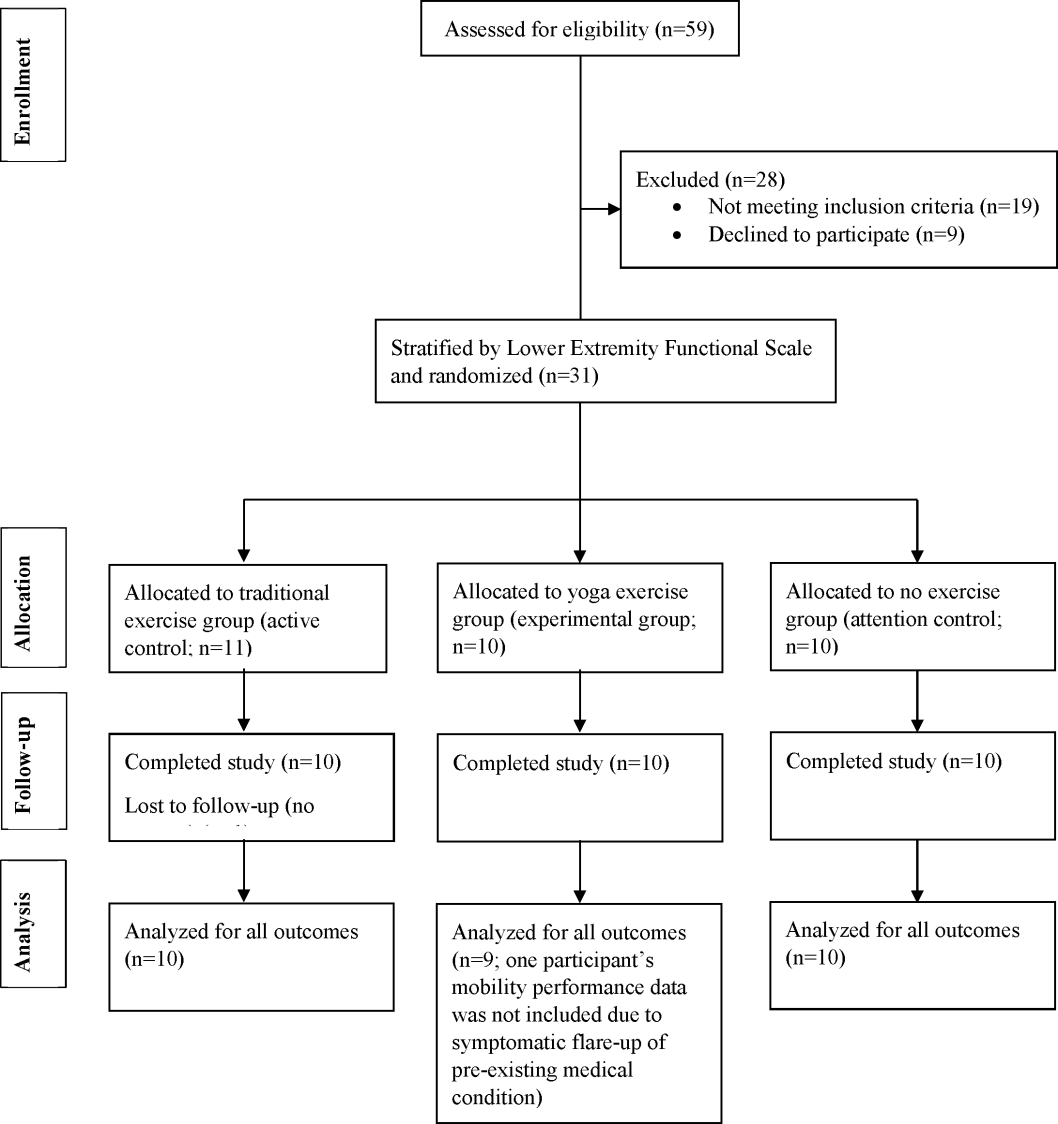
Consolidated standards of reporting trials (CONSORT) diagram of participant flow throughout recruitment, allocation, data collection and analysis.

Participant recruitment began in April 2015 and follow-up data collection was completed by September 2015. A total of 59 individuals were screened for eligibility; of these, some did not meet the inclusion criteria (n = 19), or declined to participate (n = 9). Thirty-one individuals were stratified by LEFS score and randomly allocated to either YE (n = 10), TE (n = 11), or NE (n = 10) (Table 1). One participant in TE completed only two training sessions and was lost to follow-up; therefore data from 10 participants in this group were available for per-protocol analysis. A CONSORT diagram is illustrated in Fig 1. Mean ± standard deviation session attendance was 3.0±0.75, 2.7±0.52 and 2.7±0.62 sessions per week for YE, TE, and NE, respectively. One participant in TE was unable to complete the intervention due to an unrelated health diagnosis; nonetheless, follow-up data was obtained and included in analysis. There were no significant differences in age (p = 0.17), BMI (p = 0.25), or LEFS scores (p = 0.94) between groups at baseline.

**Table 1:** Baseline clinical characteristics (expressed as mean ± standard deviation) of yoga exercise (YE), traditional exercise (TE), and no exercise (NE) groups. The number of participants that self-reported using analgesic drugs is denoted for each group.

Characteristic	YE (n = 10)	TE (n = 11)	NE (n = 10)
Age [years]	65.5±5.6	63.7±8.9	71.1±9.3
Height [m]	1.6±0.1	1.6±0.1	1.6±0.1
Body mass [kg]	75.5±7.0	74.6±21.0	83.0±17.3
Body Mass Index [kg/m^2^]	30.1±3.8	28.9±6.4	32.3±5.7
Lower Extremity Functional Scale [/80]	43.1±9.0	41.7±14.8	41.6±14.1
Currently using analgesic drugs [number of participants]	7	5	7
Number of co-morbidities*	2.1±1.0	2.1±1.7	2.9±1.4

*Co-morbidities include self-reported: heart disease, high blood pressure, lung disease, diabetes, ulcer or stomach disease, kidney disease, liver disease, anemia or other blood disease, cancer, depression, osteoarthritis/degenerative disease, back pain, rheumatoid arthritis or other conditions.

### Adverse events and co-intervention

There were no adverse events related to any of the interventions. There was one case of co-intervention. This participant (TE) received one corticosteroid and two hyaluronic acid injections in the right knee on unknown dates throughout the 12-week intervention. Data from this participant were included in analysis given this series of injections began prior to study.

### Between-group comparisons

**Fig 2:**
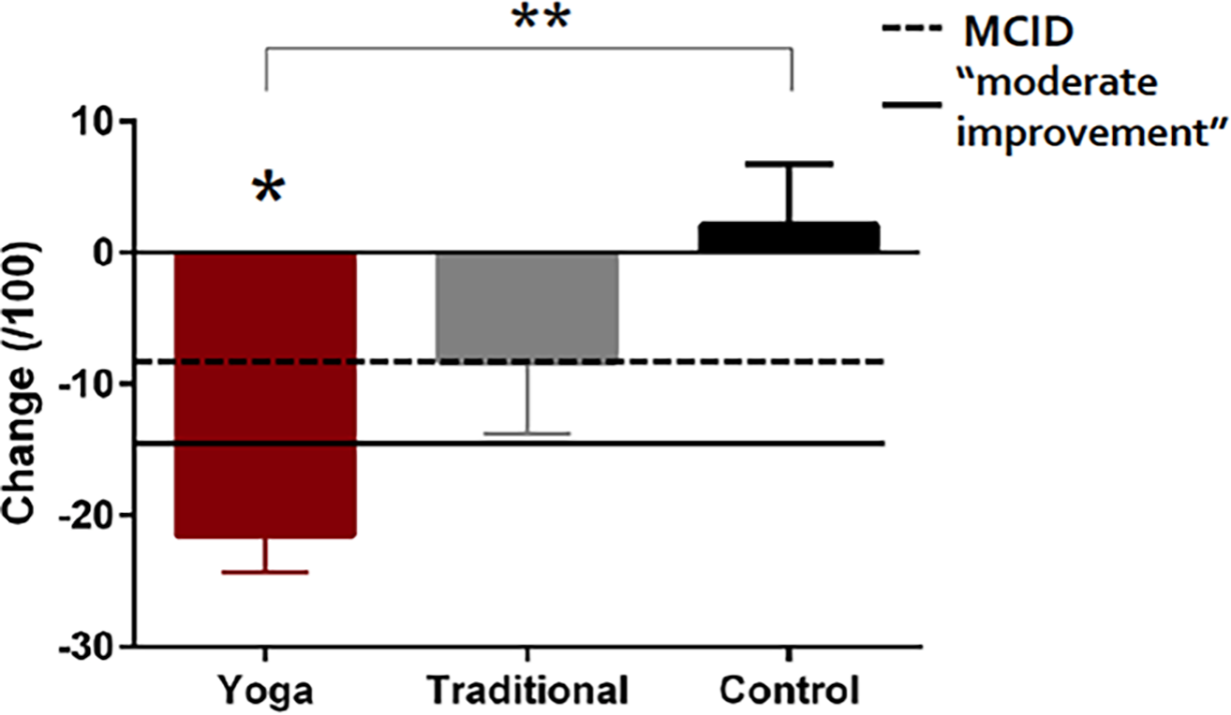
Mean ± standard error change (baseline minus follow-up values) in pain scores of the Knee Osteoarthritis Outcome Score relative to the minimal clinically important difference (MCID) and moderate improvement values. Significant between-group differences are denoted with ** and within-group change with *.

Significant differences in intervention-induced improvements between the three groups were present for certain primary and secondary outcomes (Table 2). YE demonstrated a greater improvement than NE in both KOOS pain (Table 2; Fig 2) and intermittent pain (Table 2), while differences between YE–TE, and TE–NE were not significant (Table 2; Fig 2). There were no differences in the change of constant pain between the three groups (Table 2).

**Table 2:** Pairwise between-group mean differences and percent differences in outcome measures using analysis of covariance adjusting for baseline values. Percent differences were calculated as [follow-up minus baseline]/baseline.

	YE vs. NE		YE vs. TE		TE vs. NE	
	Mean difference (YE minus NE)	P value	Mean difference (YE minus TE)	P value	Mean difference (TE minus NE)	P value
	[95% CI]		[95% CI]		[95% CI]	
	(% difference)		(% difference)		(% difference)	
**Primary Outcomes**						
KOOS Pain (/100)	22.9* [6.9, 38.8] (52.0%)	0.003	11.3 [-5.1, 27.6] (17.6%)	0.247	11.6 [-4.5, 27.7] (34.4%)	0.212
Intermittent Pain (/100)	-19.6* [-34.8, -4.38] (-43.6%)	0.009	-8.3 [-23.6, 7.0] (-19.5%)	0.448	-11.3 [-26.6, 4.0] (-24.1%)	0.448
Constant Pain (/100)	-16.0 [-39.0, 7.0] (-64.8%)	0.240	-11.8 [-34.8, 11.2] (-29.2%)	0.492	-4.2 [-27.2, 18.8] (-35.6%)	0.955
LEFS (/80)	17.2* [5.2, 29.2] (45.0%)	0.003	3.3 [-8.7, 15.3] (-4.9%)	0.870	13.9* [2.0, 25.9] (49.8%)	0.019
KOOS Function in ADL (/100)	17.9* [3.8, 32.0] (40.5%)	0.010	7.6 [-7.0, 22.2] (16.4%)	0.477	10.3 [-4.3, 24.8] (24.1%)	0.228
KOOS Function in SR (/100)	24.7 [-3.2, 52.5] (75.1%)	0.094	-6.2 [-34.1, 21.8] (-122.9%)	0.925	30.8* [3.0, 58.7] (198.0%)	0.027
Six Minute Walk (m)	24.4 [-21.6, 70.4] (13.3%)	0.463	-0.52 [-45.7, 44.7] (3.5%)	1.000	24.9 [-21.0, 70.8] (9.8%)	0.444
40 Meter Walk (s)	-1.2 [-4.4, 2.0] (-17.6%)	0.710	0.3 [-2.9, 3.4] (-2.7%)	0.995	-1.5 [-4.7, 1.7] (-14.8%)	0.573
30 Second Chair Stand (reps)	1.3 [-1.1, 3.7] (18.6%)	0.453	0.8 [1.6, 3.2] (9.8%)	0.776	0.5 [-1.9, 2.9] (8.8%)	0.937
Timed Up and Go (s)	-1.0 [-2.1, 0.1] (-13.6%)	0.074	-0.4 [-1.6, -0.7] (-9.1%)	0.756	-0.6 [-1.8, 0.6] (-4.5%)	0.487
Stair Ascent (s)	-1.4 [-3.0, 0.3] (-39.8%)	0.131	-0.4 [-2.1, 1.2] (-13.4%)	0.891	-1.0 [-2.6, 0.7] (-26.5%)	0.399
**Secondary Outcomes**						
KOOS QoL (/100)	15.2 [-2.0, 32.3] (52.0%)	0.095	4.8 [-12.8, 21.6] (-8.0%)	0.891	10.8 [-6.5, 28.1] (60.0%)	0.327
CESD (/60)	0.2 [-5.9, 6.4] (-44.2%)	1.000	-1.6 [7.6, 4.4] (-13.1%)	0.882	1.8 [-4.1, 7.7] (-31.0%)	0.829
Knee extensor strength (Nm/kg)	0.1 [-0.1, 0.3] (14.8%)	0.250	0.0 [-0.1, 0.2] (6.3%)	0.910	0.1 [-0.1, 0.2] (8.5%)	0.603
Knee flexor strength (Nm/kg)	-0.3 [-0.1, 0.1] (-3.5%)	0.791	-0.1* [-0.2, -0.1] (-28.5%)	0.028	0.1 [-0.0, 0.2] (25.0%)	0.190

A Sidak adjustment for multiple comparisons was used. Significant differences are denoted with an asterisk*.

YE = Yoga Exercise; TE = Traditional Exercise; NE = No Exercise; KOOS = Knee Osteoarthritis Outcome Score; ADL = activities of daily living; SR = sport and recreation; QoL = quality of life

Note: due to baseline values of zero, percent difference could not be calculated for certain participants and measures (KOOS Function in SR, n = 2; Constant Pain (/100), n = 5; CESD, n = 1).

**Fig 3:**
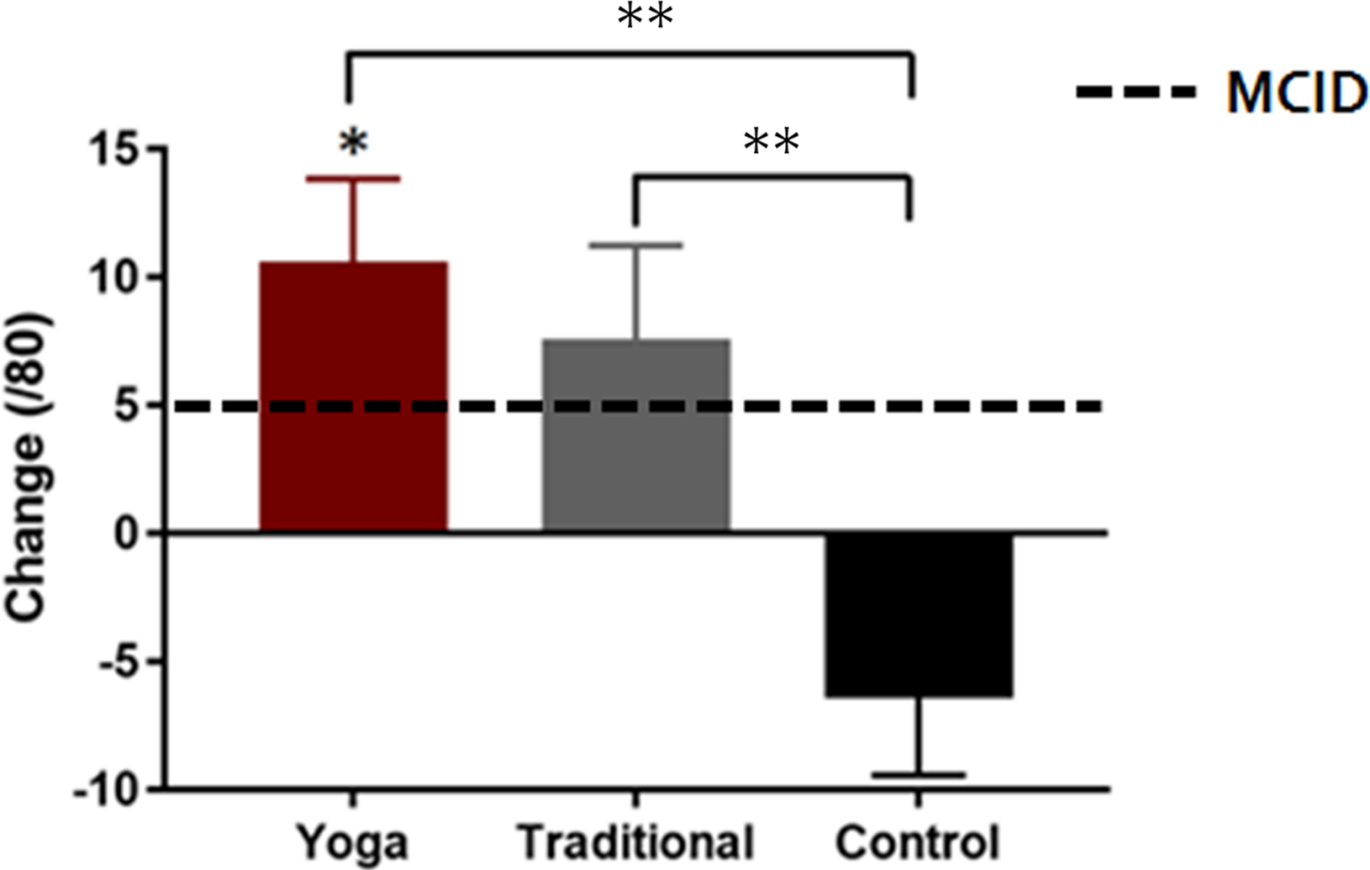
Self-reported physical function measured using the Lower Extremity Functional Scale. Data are presented as mean ± standard error change (follow-up minus baseline values) in scores relative to the minimal clinically important difference (MCID) values. Significant between-group differences are denoted with ** and within-group change with *.

**Fig 4:**
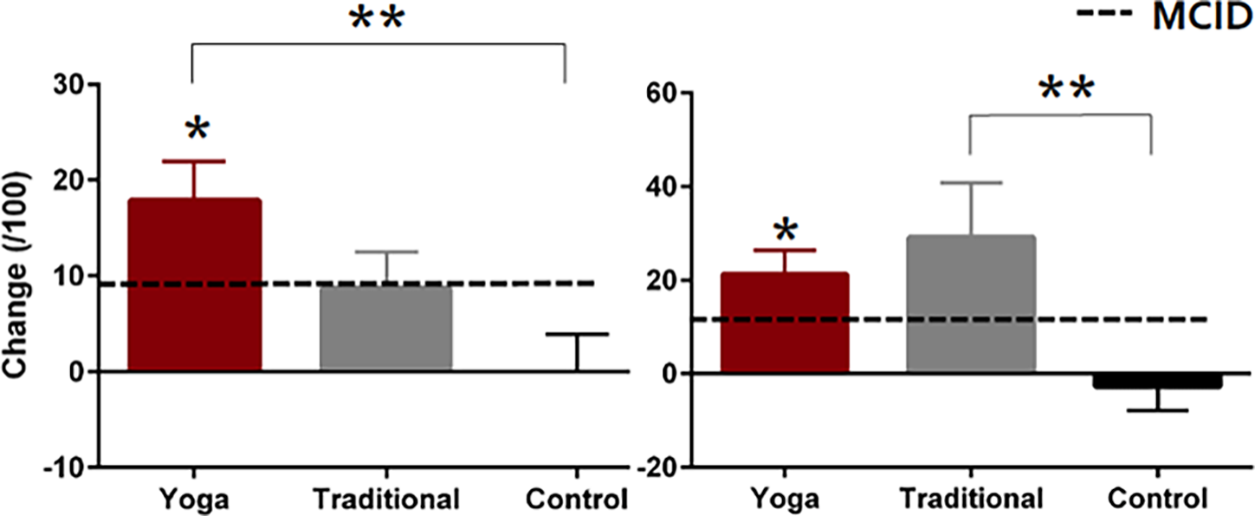
Self-reported physical function measured using the Knee Osteoarthritis Outcome Score. Data are presented as mean ± standard error change (follow-up minus baseline values) in scores relative to the minimal clinically important difference (MCID) values. Significant between-group differences are denoted with ** and within-group change with *.

Both groups performing exercise (YE and TE) demonstrated greater improvements in self-reported physical function as measured by the LEFS while there were no significant differences between the exercise conditions (Table 2; Fig 3). YE demonstrated greater improvements in KOOS ADL scores compared to NE (Table 2; Fig 4), while differences between YE–TE and TE–NE were not significant (Table 2; Fig 4). TE reported greater improvements in KOOS SR scores compared to NE (Table 2; Fig 4). The differences between YE–TE and YE–NE were not significant (Table 2, Fig 4). One participant in the TE group was removed from analyses of mobility performance measures as their data existed beyond three standard deviations from the group mean. There were no significant between-group differences in mean changes for any of the mobility performance measures (Table 2).

There was a greater increase in knee flexor strength in TE relative to YE (Table 2). There were no significant differences between groups in mean changes of KOOS QoL scores, CESD scores, or knee extensor strength (Table 2).

### Within-group comparisons

Improvements from baseline to follow-up were present for primary and tertiary outcomes in the YE and TE exercise groups (Table 3). There was an improvement in KOOS pain (Fig 2; Table 3), intermittent pain and constant pain in the YE group (Table 3). An improvement in intermittent pain was also demonstrated in the TE group (Table 3).

**Table 3:** Within-group differences in outcome measures. Baseline and follow-up group means ± standard deviations are presented with mean differences (follow-up minus baseline) and 95% confidence intervals (CI).

	Yoga exercise (n = 10)			Traditional exercise (n = 10)			No exercise (n = 10)		
	Baseline	Follow-up	Mean difference	Baseline	Follow-up	Mean difference	Baseline	Follow-up	Mean difference
			[95% CI]			[95% CI]			[95% CI]
**Primary Outcomes**									
KOOS Pain (/100)	48.8±12.4	70.3±12.8	21.5* [15.1, 27.9]	57.3±14.3	65.6±13.7	8.3 [-4.1, 20.7]	52.0±17.9	49.9±24.7	- 2.1 [-12.6, 8.4]
Intermittent Pain (/100)	52.2±14.9	28.5±16.9	- 23.7* [-30.9, -16.5]	46.4±22.2	32.1±20.6	- 14.3* [-23.4, -5.2]	52.6±27.1	48.4±27.2	- 4.2 [-16.7, 8.3]
Constant Pain (/100)	36.5±27.7	12.0±14.9	- 24.5* [-40.0, -9.0]	40.5±21.1	25.5±23.5	- 15.0 [-29.5, -0.5]	40.0±34.3	29.5±28.7	- 10.5 [-34.1, 13.1]
LEFS (/80)	43.1±9.0	53.7±12.1	10.6* [3.3, 17.9]	41.2±15.5	48.8±16.2	7.6 [-0.63, 15.8]	41.6±14.1	35.2±15.0	-6.4 [-13.2, 0.4]
KOOS Function in ADL (/100)	56.2±16.7	74.1±15.1	17.9* [8.7, 27.2]	66.0±17.6	74.7.7±16.0	8.7 [0.2, 17.2]	56.3±15.2	56.3±23.1	0.0 [-8.6, 8.6]
KOOS Function in SR (/100)	33.0.7±17.0	54.3±20.2	21.3* [9.7, 32.9]	28.5±14.7	57.7±31.1	29.2 [2.9, 55.5]	31.3±24.8	28.6±27.8	-2.7 [-14.5, 9.1]
Six Minute Walk (m)	426.9±91.9	486.19±67.0	59.3* [22.5, 96.0]	456.0±61.6	510.0±77.0	54.0* [28.5, 79.5]	428.2±78.9	447.3±108.7	19.1 [-16.8, 54.9]
40 Meter Walk (s)	33.4±7.3	29.5±3.8	-3.9 [-7.4, -0.3]	30.8±3.5	27.9±3.7	-2.9* [-5.0, -0.8]	33.5±6.3	37.5±23.6	4.01 [-9.9, 17.9]
30 Second Chair Stand (reps)	9.4±2.7	12.8±2.5	3.4* [2.0, 4.8]	10.0±3.3	12.5±4.4	2.5* [0.9, 4.1]	9.6±2.0	11.4±2.2	1.8 [0.3, 3.3]
Timed Up and Go (s)	9.0±1.4	7.7±0.7	-1.3 [-2.3, -0.3]	7.9±0.9	7.6±1.2	-0.3 [-1.0, 0.4]	9.5±1.7	9.7±3.3	0.2 [-1.2, 1.6]
Stair Ascent (s)	7.1±2.1	5.4±1.2	-1.7* [-2.8, -0.7]	6.1±1.3	5.3±1.2	-0.7* [-1.3, -0.2]	7.2±2.9	9.0±8.3	1.9 [-2.8, 6.5]
**Secondary Outcomes**									
KOOS QoL (/100)	33.8±16.5	47.7±17.0	13.9* [5.7, 22.1]	29.7±16.7	40.4±17.8	10.7 [-4.9, 26.3]	35.6±20.6	33.8±23.2	-1.8 [-9.8, 6.2]
CESD (/60)	7.6±5.1	6.6±5.6	1.0 [-4.6, 2.6]	11.3±6.9	10.3±8.2	1.0 [-3.5, 1.5]	13.2±9.8	9.6±5.7	-3.6 [-9.5, 2.3]
Knee extensor strength (Nm/kg)	0.9±0.3	1.0±0.3	0.1 [0.0, 0.2]	1.0±0.2	1.0±0.2	0.0 [-0.0, 0.1]	0.9±0.2	0.9±0.3	0.0 [-0.1, 0.1]
Knee flexor strength (Nm/kg)	0.4±0.1	0.4±0.1	0.0 [-0.1, 0.1]	0.4±0.1	0.5±0.1	0.1* [0.0, 0.2]	0.4±0.1	0.4±0.1	0.0 [-1.0, 0.1]

Significant differences compared using 2-tailed paired t-tests are denoted with an asterisk*. A Bonferroni corrected alpha value of 0.017 was used to adjust for multiple comparisons. KOOS = Knee Osteoarthritis Outcome Score; ADL = activities of daily living; SR = sport and recreation; QoL = quality of life; greater values on KOOS represent less troublesome scores (including Pain subscale)

Improvements in the LEFS, KOOS ADL and SR scores, as well as certain mobility performance measures (6MWT, 30sCS, SA) were present in the YE group (Table 3). Improvements in all mobility measures with the exception of the TUG were present in the TE group (Table 3).

With respect to secondary outcomes, YE demonstrated an increase in KOOS QoL, while TE demonstrated an increase in knee flexor strength (Table 3). No other within-group differences were present in tertiary outcomes.

No significant within-group differences from baseline to follow-up were present for any of the primary or secondary outcomes in NE (Table 3).

## Discussion

This study featured a direct comparison of a biomechanically-designed yoga program with the current gold standard of exercise, and a no exercise attention equivalent control in women with clinical knee OA. Relative to the NE control group, YE experienced greater improvements in pain and self-reported physical function. Improvements in such outcomes were similar between the YE and TE groups. These findings are consistent with the study hypothesis. This yoga program appears to be an efficacious exercise option that is comparable, and in some aspects, potentially superior to traditional exercise for alleviating the physically debilitating symptoms of knee OA in women.

### Strengths and limitations

An important feature was the randomized control trial design including an experimental group, an active treatment comparison group, and an attention-equivalent control group. However, based on a-priori power calculations this study was underpowered. We aimed to recruit 60 individuals; however of the 59 individuals we screened for participation, only 31 met the eligibility criteria and agreed to participate. We did not extend our recruitment period to boost our sample size to ensure that the group interventions began in a timely manner from the time of enrolment. Any further delay in commencing the intervention may have resulted in participant drop-out. As a result of this limited sample, post-hoc analyses revealed observed power values for the primary outcome measures between 0.32 and 0.81, with all but one over 0.79. We acknowledge this small sample is a key limitation. In future work, we aim to conduct a larger, multi-site trial to boost our sample size. However, despite this limited sample, important improvements in pain and physical function were elicited, demonstrating the importance of exercise for older adults with knee OA. Another limitation is the use of multiple outcome measures. Knee OA produces multiple sequelae for the person with disease. To capture the breadth of disease impact as recommended in OA [27–30], we measured pain, self-reported physical function and mobility performance as primary outcomes. While these measurements provide insight on the breadth of impact of the interventions, the key focus to improve patient care should be on pain. As such, pain was used to estimate sample size. Also, due to this small sample size, we were unable to analyze sub-groups (such as those stratified by age, BMI or disease severity), and therefore unable to speculate whether the interventions had a greater effect in some individuals compared to others. These sub-analyzes would be important to consider in future work. As well, analyses of cartilage morphology, inflammatory markers and biomechanical analyses were removed because several participants did not consent to and/or complete these measurements at baseline and follow-up; and cardiovascular fitness and muscle/fat volume were not measured due to inadequate funding. Further, it is important to consider that while exercises included in the YE intervention were those that imposed a negligible KAM, knee joint loading incurred during the exercises included in the TE program were not measured.

### Clinical relevance of findings

The clinical relevance of the measured outcomes was interpreted using MCID and PASS values. The MCID of LEFS is 5 points [43] and the MCID for KOOS scores is a change of 8 to 10 points [1]. In this sample, clinically important improvements in LEFS scores of 10.6 and 7.6, as well as KOOS pain scores of 21.5 and 8.3, were observed in the YE and TE groups, respectively. A change of 15 points is indicative of “moderate improvement” for KOOS pain [44]. In the YE group, all 10 participants demonstrated improvements in pain scores above the MCID with 8/10 demonstrating a moderate improvement (≥15 points). For TE and NE groups, 5/10 and 3/10 participants had improvements above the MCID. For the LEFS, 6/10, 5/10 and 2/10 participants had clinically important improvements in the YE, TE and NE groups, respectively.

Improvements in intermittent and constant pain subscales of the ICOAP met the MCID in YE only [42,45]. The PASS values for the ICOAP are reportedly 40 for intermittent pain and 20 for constant pain [42,45]. At baseline, all three groups reported intermittent and constant pain scores greater than PASS values; indicating unacceptable levels of pain. At follow-up, intermittent pain scores were less than the PASS value in the YE and TE groups, while for constant pain, only the YE group attained a mean score less than the PASS value. Examining individual scores, in the YE group 7/10 and 8/10 participants had constant and intermittent ICOAP scores that were less than the PASS value, indicating pain intensity was likely considered acceptable to the participant. This compared to 4/10 and 8/10 for the TE group and 4/10 and 5/10 for the NE group for constant and intermittent scores, respectively. Regarding quality of life, YE and TE met the MCID in the KOOS ADL scores (Fig 4). For KOOS SR scores, clinically important improvements of 21.3 and 29.2 were observed in the YE and TE groups, respectively (Fig 4) [44]. A non-clinically relevant decrease of 2.7 was observed in the NE group [44].

There are no established MCID values for mobility performance measures after an exercise intervention among those with knee OA. In OA of the hip, an increase of 2 to 3 repetitions in the 30sCS test and a decrease of 0.8 to 1.4 seconds in the TUG test have been considered clinically important [35]. In the current study, the YE and TE groups performed an additional 3.4 and 2.5 repetitions in the 30sCS test, respectively; and decreased TUG times by 1.4 and 0.3 seconds, respectively.

Conceptually, the MCID represents improvement associated with “feeling better;” whereas the PASS is designed to reflect partial symptomatic remission, relating to “feeling good” [42]. From a clinical standpoint, the YE group appeared to achieve more meaningful improvements in pain and self-reported physical function compared to the TE and NE groups. However, despite demonstrating larger improvements in pain and function in the YE group compared to the TE group, there were no significant differences between the interventions. This finding may seem surprising because the YE group received an intervention combining mindfulness and biomechanical exercise, compared to TE which received exercise alone. However, because that the intervention focussing on mindfulness alone (NE group) yielded no meaningful benefits, it seems reasonable that the mindfulness element of YE yielded no benefit above that of physical exercise. The absence of significant differences between groups may also be attributed to the small sample size in each group given the large variability present in the outcome measures. It is also possible that the exercises performed in the TE program had imparted comparable joint loads to the YE program. In the future, it would be interesting to conduct biomechanical analysis of the exercises included in the TE program in addition to the YE program.

### Congruence with previous studies

The effectiveness of land-based exercise for improving pain, physical function, and quality of life is well-established. A large-scale review of 44 studies demonstrated high quality evidence that land-based exercise yields a 12% (95% CI, 10–15) absolute and 27% (95% CI, 21–32) relative improvement in pain for individuals with knee OA [2]. This meta-analysis also included 44 studies providing moderate quality evidence suggesting a 10% (95% CI, 8–13) absolute and 26% (95% CI, 20–32) relative improvement in physical function for those who engage in an exercise intervention. Lastly, there was high quality evidence that a 4% (95% CI, 2–5) absolute and 9% (95% CI, 5–13) relative improvement could be expected in quality of life following an exercise program. The findings of the current study are consistent with this evidence. Pain (ICOAP total score), a 24% absolute and 53% relative improvement was observed in YE. There was a 15% absolute and 34% relative improvement in TE; and a 4% absolute and 8% relative improvement in NE. These trends were similar for secondary outcomes self-reported physical function and mobility performance. The TE intervention in this study was designed to reflect the current exercise prescription for knee OA, which was very similar to the majority of the studies reviewed in the meta-analysis [2]. Not surprisingly, the magnitude of change in pain, self-reported function, and quality of life in the TE group was similar to those values presented in the review, in some cases slightly larger. The magnitude of improvement in the YE group was substantially larger however. Improvements in pain and mobility performance also exceeded those reported in a recent study, which compared changes in self-reported and objective measures following either a 12-week leg-based or hip-based strengthening program in older adults with knee OA [46]. In the leg-based exercise program, KOOS pain improved a mean of 14.95 (95% CI, 9.34 to 20.56; p<0.01) which was a smaller improvement than that seen in the YE group (mean, 21.5; p<0.017), while larger than that in the TE group (mean difference, 21.5; p<0.017) in the current study. However, improvements in mobility performance, particularly the 6MWT were larger in both YE (mean difference, 59.3 m; p<0.017) and TE (mean difference, 54.0 m; p<0.017) groups compared to this recent research (mean difference, 26.0 m; N.S.). This improvement in the 6MWT distance was also larger than those reported in another recent study that compared 6 weeks of low intensity supervised (mean difference, 30 m; p = 0.007) versus home-based (mean difference, 30 m; p = 0.022) strengthening exercises in a population with knee OA [47].

The improvements in pain, self-reported physical function, and mobility performance in the YE group are consistent with our previous work and other studies investigating yoga for OA [10,17,18]. However, there is ample evidence, including our previous cohort study [10], that strength increases are achievable in knee OA [48]. Thus, it was surprising there were no significant improvements in the YE group. Lower extremity muscle strength, especially that of the quadriceps, is an important topic in knee OA rehabilitation [49] and is of great interest to researchers. It is not however what individuals living with knee OA find most troubling; the pain and disability associated with the disease is of greater concern [50,51]. These data provide evidence that increases in muscle strength are not necessary to elicit improvements in symptoms of knee OA.

### Contribution to the literature

This trial and the previous cohort study [10] are the first exercise interventions designed and tested specifically to minimize potentially harmful biomechanical loads incurred by the OA affected knee joint. Previous exercise prescriptions have not been designed using measures of knee mechanics known to influence knee OA progression. We now have concrete evidence that, in those with knee OA, exposure to large KAMs is linked with increased degradation of joint tissues [5–8]. This is a concern because exercise is effective in ameliorating symptoms with regular, chronic engagement, not on an acute basis. To ensure individuals with knee OA are not compromising their joint health with exercise, the mechanical loading aspect of such physical activity cannot be disregarded. Though small, this is the first RCT to conduct a direct comparison of traditional exercise to a program designed to minimize KAM exposure.

## Conclusions

This biomechanically-based yoga intervention appears to be well tolerated and shows promise as an efficacious approach to alleviate the major burdening symptoms of clinical knee OA in women. Yoga, as delivered here, appears to be similarly efficacious to traditional exercise for improving pain, self-reported physical function, and quality of life. However, while the yoga program demonstrated benefits for pain and self-reported function compared to no exercise, it did not elicit improvements in objective measures, namely strength and mobility performance in the between-group analyses. Further, given that the yoga program did not outperform the traditional program in the outcomes measures recorded in this sample, future trials of yoga for knee OA with larger samples are warranted to establish effectiveness; as well as investigate superiority and less harm relative to traditional modes of exercise.

## Supporting information

S1 Checklist CONSORT 2010 checklist of information to include when reporting a randomised trial. (DOC)

S1 Protocol Study protocol. (DOCX)
