# Supplementary material for: Efficacy of a biomechanically-based yoga exercise program in knee osteoarthritis: A randomized controlled trial
Source: PLoS One. 2018 Apr 17;13(4):e0195653. doi: 10.1371/journal.pone.0195653 (PMC5903657; doi:10.1371/journal.pone.0195653)
Supplement: S1 Protocol — (DOCX) [file pone.0195653.s002.docx]

**S1 Protocol. Clinical and Tissue Outcomes of a Biomechanics Exercise Program**

**for Knee Osteoarthritis - Protocol**

**Introduction**

Over 4 million Canadians endure a form of arthritis^1^. This number will escalate due to the aging population and the prevalence of obesity^1^. Arthritis is a great cause of chronic disability among community-dwelling older adults^2^. The most common arthritis, osteoarthritis (OA), affects 10% of Canadian adults^1^. Osteoarthritis results from excessive and abnormal loads that degrade cartilage and bone, most frequently in the knee. Adding to the complexity of treatment, knee OA rarely occurs in isolation. This population has elevated incidences of cardiovascular, gastrointestinal disorders, and depression^1^.

Keeping older adults with knee OA moving is important, however implementation is complicated. Systematic reviews and clinical practice guidelines confirm that exercise is critical in knee OA treatment^3–5^. Exercise reduces symptoms, co-morbidity and improves physical function^3–5^. In fact, the effects of aerobic and strengthening exercise on pain are equivalent to medication^5^. However, important caveats exist. Generic exercise prescription in large samples produces mediocre findings in terms of pain and physical function^3^. It is important to note that these exercise programs did not benefit from data that would properly dose the type, intensity, repetition and biomechanical load of the exercise for people with knee OA.

There are concerns that improperly dosed exercise can accelerate knee OA progression. High loading repetition, a feature of walking programs for example, is linked to greater intensity knee pain in people with knee OA^6^. Exercise programs that require footwear may not be ideal because shoes can elevate the mechanical loads within the knee. In particular, the knee adduction moment (KAM) was increased by 38% when 68 subjects were wearing running shoes compared to barefoot^7^. The KAM reflects the proportion of load placed on the medial knee compartment that is most commonly degraded in this disease. Longitudinal studies clearly demonstrate that KAM predicts progression noted on radiographs^8^ and on magnetic resonance imaging scans^9^. Finally, while quadriceps strengthening improved symptoms in many samples with knee OA, concerns have been raised about the potential for traditional strengthening programs to increase the contact forces that may contribute to acceleration of knee OA^10,11^. These experts recommend that strengthening exercises must elicit muscle activation patterns, while maintaining ideal alignment of the lower extremity to minimize KAM.

Studies showing that generic exercise programs improved pain and mobility in people with knee OA provide confidence that exercise is fundamental to the management of this disease. Improving exercise programs to minimize biomechanical risks specific to knee OA may enhance the benefits of exercise for this population. Yoga offers a foundation of static postures that improve muscle strength and joint flexibility, while eliminating repetition, footwear and, if properly analyzed, KAM.

Yoga is an exercise activity that emphasizes a variety of static postures. Many yoga postures challenge and strengthen musculature of the lower extremity, while minimizing some potential negative effects that other exercise programs pose for people with knee OA. For example, running is an activity that includes large and repetitive loads on the knee, which may not be the best-suited activity for individuals with knee OA. Yoga has beneficial effects on various aspects of quality of life, including improved cardiorespiratory health, body awareness, well-being and mental health. Further, yoga improved strength, balance, flexibility, pain, and disability in older adults and individuals with knee OA^12–16^. For example, a pilot study consisting of 7 women over age 50 years with symptomatic knee OA implemented an 8-week program of modified yoga postures. Each week participants attended one 90-minute weekly class. Participants reported improvements in knee pain, physical function and mental health, demonstrating that yoga holds great potential as a treatment option for older, yoga-naive women with knee OA^12^.

We recently completed a pilot project (REB#13-510) that examined the efficacy of a yoga program for older women with symptomatic knee OA^13^. Primary measures in this study included self-reported pain and function, mobility performance, and strength. Secondary measures included KAM, muscle activation patterns, and muscle co-activations to ensure postures chosen (pre-determined from previous work in our lab from young healthy women^14^) did not overload the medial knee while requiring enough muscle activity to result in strengthening. We observed that 12-weeks of yoga improved self-report measures, mobility on the six-minute walk test and 30-second chair stand test, and knee extensor and flexor strength on the dynamometer^13^. We also noted that squatting and lunging postures from the yoga program exhibited low KAM (lower than that experienced during normal gait) and muscle activations appropriate for improving muscle strength^13^. The results from this study provide a rationale for continuing research on the efficacy of a biomechanical exercise program on knee OA. Specifically, it is important to note whether the improvements observed are as effective or superior to those experienced during traditional exercise prescription for knee OA via a randomized controlled trial design.

Research Question & Hypotheses

The primary objective of this study is to compare the efficacy of a biomechanical exercise program with traditional exercise and no exercise in improving clinical and tissue outcomes. We aim to determine the extent to which tissue changes explain changes in clinical outcomes as a result of the biomechanical exercise program, over and above age, psychosocial factors, and body mass index. A secondary objective is to compare the effect of the biomechanical exercise program, traditional exercise, and no exercise on KAM and muscle activation amplitude.

We hypothesize that study participants will experience improvements in self-reported outcomes, mobility, strength in the musculature surrounding the knee, muscle volume, and cartilage composition following the completion of both exercise programs, with the yoga intervention providing larger improvements for all outcomes. We also hypothesize that KAM during yoga and exercise postures will remain lower than that of gait, and muscle activations will be appropriate to stimulate muscle strength gain in the exercise groups.

**Study Design and Methods**

This study is a randomized controlled trial (RCT). Participants will be randomized into one of three study arms: biomechanical exercise program (BE), traditional exercise program (TE) and an attention-equivalent control group receiving only meditation (M). Outcome measures will be assessed at McMaster University before and after their respective 12 week intervention. The BE intervention will be delivered at a yoga studio in downtown Hamilton, Ontario. The TE intervention will be delivered at the Physical Activity Centre for Excellence (PACE) at McMaster University. Finally, the M group will receive meditation training at the same yoga studio as the BE program.

Participants

Sixty community-dwelling women, 50 years of age and older, who meet the American College of Rheumatology (ACR) criteria for clinical knee OA will be recruited to participate in this study. The ACR clinical criteria require 3 of the following:

- Knee pain on most days of the week,
- Less than 30 minutes of morning stiffness,
- Crepitus with active range of motion,
- Bony enlargement,
- Bony tenderness to palpation, and
- Signs of inflammation (warmth, swelling).

*Inclusion and Exclusion Criteria*

Women will be recruited to participate in this study. Women were selected because incidence of knee OA is two times greater among women than men over the age of 50 years^15–17^. Muscle weakness is of particular concern in women with knee OA because their absolute muscle strength is lower compared to men, and therefore women are closer to the critical thresholds of strength required for activities of daily living^18^. Finally, women report pain secondary to knee OA more frequently than men^17^ perhaps because the pain experience is different^19^. The incidence and prevalence of knee OA increases with age, particularly in women^15,17,20–22^.

To confirm radiographic OA consistent with American College of Rheumatology criteria^23^, coronal weight-bearing knee radiographs will be obtained in a standardized fixed-flexion position using a Synaflexer^TM 24^. This frame reliably places the feet in 5° of external rotation and 20° of knee flexion^24^. As well, skyline views of the patellofemoral joint will be included to ensure no pathology is present in this joint. These radiographs will be completed at Imagus Imaging Center at 25 Charlton Street, Hamilton (across the street from St. Joseph’s Hospital).

The exclusion criteria for participation include a self-reported history of patellofemoral symptoms. The exercise intervention is focused on unloading the medial knee compartment; however these exercises could place large loads on the patellofemoral joint, such as squats and lunges. In addition, participants that self-report fractures due to osteoporosis will be excluded. Exclusion criteria include diagnosis of other forms of arthritis (e.g., rheumatoid, psoriatic); active non-arthritic knee disease (e.g., gout); conditions that might be exacerbated by the protocol (e.g., unstable angina); neurological conditions such as a stroke; and current/past use of intra-articular therapies or knee surgeries and; contraindications to magnetic resonance imaging (e.g., pacemaker). Participants will be excluded if they have a skin allergy to medical tape, use an adaptive aid such as a cane or cannot climb 2 flights of stairs safely. Lower extremity trauma within 3 months, ipsilateral hip or ankle conditions, radiation (e.g., cancer treatment) and pregnancy will also be exclusion criteria.

*Recruitment*

Several methods of recruitment will be used. First, participants will be recruited through a list of potential participants provided by Dr. Rick Adachi at the Centre for Appendicular Magnetic Resonance Imaging (CAMRIS) at St. Joseph’s Hospital. All of the individuals on this list have signed a consent form to be contacted for research studies. These potential participants will be identified by a receptionist at CAMRIS and a letter will be sent by the receptionist on behalf of CAMRIS inviting their participation. The letter will invite participants to email or call a research assistant if interested in the study. A research assistant will respond to potential participants using the same mode of communication. Once in contact, the research assistant will describe the purpose, protocol, risks and benefits of the study. Potential participants who express an interest will be screened for the inclusion and exclusion criteria.

Second, posters will be displayed in the Hamilton, Ontario community, including at the yoga studio. As well, the poster will be included in weekly electronic newsletters sent out by the yoga studio. The advertisement will invite potential participants to contact a research assistant via telephone or email. A research assistant will respond to potential participants using the same mode of communication. Once in contact, the research assistant will describe the purpose, protocol, risks and benefits of the study. Potential participants who express an interest will be screened for the inclusion and exclusion criteria. Dr. Rick Adachi will first meet with the potential participant at CAMRIS to confirm the presence of clinical knee OA.

Third, the local newspaper, The Hamilton Spectator, printed an article about our yoga pilot study. A list of 53 interested participants was generated for future contact. These participants will be contacted via the same mode they contacted the Research Assistant with. Once in contact, the research assistant will describe the purpose, protocol, risks and benefits of the study. Potential participants who express an interest will be screened for the inclusion and exclusion criteria. Dr. Rick Adachi will first meet with the potential participant at CAMRIS to confirm the presence of clinical knee OA.

For all recruitment strategies, any potential participant that expresses interest and meets the criteria for the study will be sent a copy of the consent form (electronic or hard copy at the potential participant’s discretion) and a map directing them to pre-paid parking. An appointment will be set at the MacMobilize Laboratory, McMaster University. At this time, participants will be instructed to avoid any high intensity physical activity for 24 hours prior to testing.

Biomechanical Exercise (BE) Intervention

The biomechanical exercise intervention will be a 12-week yoga program requesting attendance at 3 supervised classes each week. Over each week, 4 classes will be available (Monday and Wednesday 7-8am; Friday and Saturday 3-4pm). We aim to start March 2015 to prevent the participants from commuting in the heavy snow.

This class will be conducted at a yoga studio near downtown Hamilton in a regular temperature room (De La Sol Yoga located at the corner of York and Locke Streets; http://www.delasolyoga.com). The class will be instructed by Andre Grandbois and Stephanie Miles. Both are certified yoga instructors with experience delivering the biomechanical program we are studying. The yoga studio is a commercial space with dedicated free parking, yoga mats and other equipment, bathroom facilities including showers, and an elevator. A structured fee is provided to the instructor and yoga studio to access space, parking and equipment. The participants will incur no costs associated with attending the yoga classes.

Each class will be 1 hour in length. The yoga class will include a warm-up period featuring large body movements with no resistance. Then the instructor will lead the class through a series of standing postures identified with our previous work as requiring high muscle activity from the knee musculature, but minimal knee adduction moment. Examples of these postures include squats, lunges, wide-legged stances in various ranges of knee motion, and balance poses. The instructor will offer a variety of modifications for each pose, to ensure that the participants have an alternative best suited for their experience level. In addition, a series of seated poses that focus on flexibility will be completed. Each class will conclude after a guided deep-breathing and meditation activity. Throughout the class, education about knee joint anatomy, osteoarthritis and proper lower extremity alignment will be included.

Retention strategies to enhance adherence to the exercise intervention will include the following:

- Exercise logbook to track activity;
- $50 stipend upon completion of the final data collection;
- Draw for $5 Tim Horton’s gift certificate at the conclusion of some classes; and
- Rewards for best attendance across 12-weeks including a 3 month yoga pass, yoga mats, and water bottles.

Traditional Exercise (TE) Program

The traditional exercise intervention will be a 12-week program requesting attendance at 3 classes each week. Over each week, 4 classes will be available (times TBD). We aim to start March 2015 (in parallel with the BE program) to prevent the participants from commuting in the heavy snow.

This program will be conducted at the PACE at McMaster University. The participants will follow an exercise program often prescribed for people with knee osteoarthritis. An experienced, trained Kinesiologist at the PACE will be available for providing structure to the program and encouraging program progression. The PACE is a commercial space with exercise equipment, a small walking track, free weights and bands, bathroom facilities including showers, and an elevator. A structured fee will be provided to the PACE to access space, parking, and equipment. The participants will incur no costs associated with attending the classes.

All participants will receive a workout program that will be kept in a locked cabinet at the PACE. Participants will be asked to follow the program to the best of their ability, and to seek assistance from the trained Kinesiologist if needed. Each session will include a warm-up period featuring large body movements with no resistance. Then the workout will include a series of traditional exercises prescribed for knee OA including cardiovascular training (walking), single leg balance, muscle strengthening machines, and band work. The Kinesiologist will be available to offer a variety of modifications for each exercise to ensure that the participants have an alternative best suited for their experience level. In addition, a series of flexibility exercises will be included in a cool-down section at the end of each session.

Retention strategies to enhance adherence to the exercise intervention will include the following:

- Exercise logbook to track activity;
- $50 stipend upon completion of the final data collection;
- Draw for $5 Tim Horton’s gift certificate at the conclusion of some visits; and
- Rewards for best attendance across 12-weeks including movie passes and water bottles.

Attention Control (M) Program

The attention control intervention will be a 12-week meditation and mindfulness program requesting attendance at 3 supervised classes each week. Over each week, 4 classes will be available (times TBD). We aim to start March 2015 (in parallel with the BE and TE programs) to prevent the participants from commuting in the heavy snow.

This class will be conducted at a yoga studio near downtown Hamilton in a regular temperature room (De La Sol Yoga located at the corner of York and Locke Streets; <http://www.delasolyoga.com>). The class will be instructed by a certified yoga instructor specialized in meditation. The yoga studio is a commercial space with dedicated free parking, yoga mats and other equipment, bathroom facilities including showers, and an elevator. A structured fee is provided to the instructor and yoga studio to access space, parking and equipment. The participants will incur no costs associated with attending the yoga classes.

Each class will be 1 hour in length. These classes will focus on body awareness, relaxation, and mindfulness exercises associated with deep breathing. These classes will not include any physical exercise.

Retention strategies to enhance adherence to the exercise intervention will include the following:

- Exercise logbook to track activity;
- $50 stipend upon completion of the final data collection;
- Draw for $5 Tim Horton’s gift certificate at the conclusion of some classes; and
- Rewards for best attendance across 12-weeks including movie passes and water bottles.
- Offer of a monthly pass (up to 3 months are available if participants attend at least 5 sessions per month) to the PACE to access the traditional exercise program upon completion of the follow-up measurements.

Outcome Measures

Outcome measures will be collected at baseline and after completion of the respective 12-week programs. These outcome measures will be collected at the MacMobilize Laboratory (Communications Research Laboratory Building, room B110) on McMaster campus. A study limb will be chosen as the most symptomatic knee. For the TE and BE groups, baseline and post-intervention MRIs will be collected at the Imaging Research Centre at St. Joseph’s Hospital.

*Primary Outcome Measures*

*A. Self-Reported Outcomes*

- Lower Extremity Functional Scale (LEFS)
  - Functional limitations will be quantified on the Lower Extremity Functional Scale (LEFS), a 20-item self-report measure validated for knee OA where better function is represented by a higher score out of 80^25–27^. The LEFS consists of 20 items, on an adjectival scale, that assess difficulty during mobility tasks ranging from transfers to running. This range avoids ceiling and floor effects. Higher scores represent better self-reported physical function. It is reliable and valid in knee OA and has superior sensitivity to change compared to similar measures^25,28,29^.
- Numeric Pain Rating Scale (NPRS)
  - Participants will be asked to rate the intensity of their knee pain on the Numeric Pain Rating Scale (NPRS) following completion of knee extension and flexion efforts, as well as following the stair climbing protocol. The self-reported measure consists of an 11-point scale (0 to 10) with 0 indicating “no pain” and 10 representing “pain as bad as can be.” Participants will be asked to rate their pain intensity from the activity. These 2 scores will be presented out of a maximum score of 10. The NPRS has been used as a subjective measurement of pain in individuals with orthopedic dysfunction of the lower extremity^30,31^. The NPRS has demonstrated a test-retest reliability coefficient of 0.67 – 0.97^32–34^. With respect to convergent cross-sectional construct, the NPRS has been compared with the visual analogue scale resulting in correlation coefficients of 0.79 – 0.95^35^. High within-subject confidence in measured score ratings have been reported with the NPRS, demonstrated by the 90% confidence interval equaling ± 2 points on the scale^31^.
- Knee injury and Osteoarthritis Outcome Score (KOOS)
  - The KOOS is a patient-administered, 42 item questionnaire.  Study participants are asked to answer questions on a 5-point Likert scale, which takes approximately 10 minutes to complete.  The KOOS consists of 5 subscales:  pain, other symptoms, activities of daily living (ADL), function in sport and recreation and knee-related quality of life (QoL).  The questionnaire results in a normalized score out of 100 for each subscale, where 100 indicates no symptoms and 0 indicates extreme symptoms. The questionnaire relies on subject’s recall from the previous week.

The KOOS has been used in persons 13-79 years of age. The KOOS reference values are available from various sample populations including; age/sex based samples^36,37^, active soccer players^38^, and male and female patients post anterior cruciate ligament reconstruction^39^. The KOOS has a high test-retest reliability demonstrated by intra-class correlation coefficients of 0.80-0.97 for the Pain subscale, 0.74-0.94 for the Symptom subscale, 0.84-0.94 for the ADL subscale, 0.65-0.92 for the Sport/Rec subscale and 0.60-0.91 for the QOL subscale in patients with knee osteoarthritis (OA)^40^. The minimal detectable change values for patients with knee OA are 13.4 for Pain, 15.5 for Symptoms, 15.4 for ADL, 19.6 for Sport/Rec, and 21.1 for QOL^41^. Currently, the minimal clinically important change value is suggested to be 8-10 for the KOOS, proving the scale is sensitive to change^41^. The KOOS has been reported to detect change following surgical procedures including; ACL reconstruction, meniscectomy, cartilage repair procedures, tibial osteotomy, total knee replacement and non-surgical procedures including; physical therapy, as well as nutritional and pharmaceutical interventions^42^. The KOOS presents an internal consistency range (Cronbach’s α) of 0.65–0.94 for Pain, 0.56–0.83 for Symptoms, 0.78–0.97 for ADL, 0.84–0.98 for Sport/Rec and 0.71–0.85 for QOL in patients with knee OA^41^. The KOOS has demonstrated convergent and divergent construct validity when compared against numerous instruments including the different subscales of the Short Form-36 and the Lysholm knee scoring scale^41^.

The KOOS includes the complete and original WOMAC OA Index LK 3.0 therefore WOMAC scores can be calculated in isolation. The KOOS subscales ‘Sport and Recreation function’ and ‘Quality of Life’ have been shown to be more sensitive and distinct than the WOMAC subscales ‘Pain’, ‘Stiffness’, and ‘Function’ when studied in subjects with radiographic evidence of OA who underwent a meniscectomy 21 years prior to the study (mean age = 57 years, range = 38-76) compared to age and gender matched controls^37^.

- Measure of Intermittent and Constant Osteoarthritis Pain (ICOAP)
  - ICOAP is a relatively new patient-administered questionnaire.  The ICOAP was derived from focus group findings regarding the pain experience of people with knee OA^43,44^.  To complete the questionnaire, which takes under 10 minutes, study participants are asked to answer 11 questions on a 5-point Likert scale.  Higher scores are indicative of more severe pain. This questionnaire asks study participants to comment on the intermittent and constant symptoms of their worst or most troublesome knee joint and includes questions regarding intensity, frequency and impact on mood, sleep and quality of life, independent of the effect of pain on physical function.  The questionnaire relies on subject’s recall from the past week.
  - The ICOAP produces scores that are reliable and valid for use among adults with knee OA^43^.   With respect to content validity, Cronbach’s α value was reported to be 0.93 in 100 individuals with knee or hip OA^43^.  Regarding test-retest reliability, an intraclass correlation coefficient of 0.85 (95% confidence interval 0.76-0.91) was reported in 76 individuals; age 40 years or greater, with knee or hip OA^43^.  Total and subscale ICOAP scores are significantly correlated with scores from the Western Ontario and McMaster Universities Osteoarthritis Index (WOMAC) pain scale, the Knee Injury and OA Outcome Score (KOOS) symptoms scale, and self-rated effect of hip/knee problems on quality of life using the Spearman’s correlation coefficients with values ranging from 0.60 (KOOS symptoms) to 0.81 (WOMAC pain scale)^43^.  The ICOAP has been demonstrated to detect changes in OA pain due to pharmacological interventions^43^ and joint replacement surgery^45^.  Scores are produced separately (for the distinct pain types) and a total score can be obtained by summing the scores and then normalizing from 0 (no pain) to 100 (extreme pain).

*B. Mobility Performance Measures*

The mobility performance measures selected reflect the core set of mobility measures recommended by the Osteoarthritis Research Symposium International (OARSI)^54^.

- Six-Minute Walk Test (6MWT)
  - The 6MWT is used to quantify walking ability. The 6MWT is an inexpensive clinical tool that involves recording the distance that participants cover while walking indoors at their own pace for 6 minutes. Participants are free to stop or use a mobility aid to complete the walking task, making this measure clinically useful. The 6MWT measurement will be recorded indoors in a well-lit, tiled rectangular hallway. The score recorded is the total distance traveled in 6 minutes. Instructions for the 6MWT will correspond with the published protocol^55^. The 6MWT yields highly reliable (intraclass correlation coefficient=.96) and valid data^56^.
- 40-metre Fast Paced Walk
  - The 40-metre walk will be measured in conjunction with the 6MWT. Participants will be provided with the same instructions as those presented for the 6MWT. Briefly, participants will be asked to walk around a well-lit, rectangular hallway at their own pace for 6 minutes. Participants will be timed with a stopwatch and distance covered will be measured with an odometer wheel. The first 40-metres of the walk (marked by coloured tape marks on the floor and measured with the odometer) will be timed by the researcher. The participant will be asked to continue the 6MWT protocol and the time to reach 40-metres will be recorded.
- Stair Climbing
  - Participants will be asked to ascend and descend a 9-step staircase as quickly as possible, without compromising their safety. The hand rail can be used as needed. The participants will be asked to start at the base of the stairs at a distance from the first step of their choosing. The participants will be instructed not to run or jog, and not to skip any steps. Time to ascend and descend will be recorded separately, to the nearest 10^th^ of a second, using a stopwatch. Participants will be asked to repeat this procedure two times, where scores from only the ascent and descent on the second attempt are recorded. Data from our laboratory demonstrate excellent test-retest reliability of the stair ascent and descent tasks among 29 adults (ICC=0.881, 0.843 respectively).
- 30-Second Chair Stand Test
  - The 30 second chair stand task quantifies the number of sit-to-stand movements that can be completed within 30 seconds, starting from a seated position in an armless chair of a standard height (45 cm). After an opportunity to practice the task, participants will be asked to perform this task at a comfortable pace. Knee extensor strength is a strong predictor of performance on repeated chair stand tests^57^. In addition, lower extremity muscle power was predicted from performance of the 30 second chair stand in 14 older adults^58^.
- Timed Up and Go (TUG)

The TUG evaluates the time it takes a participant to rise from a chair, walk 3 metres, turn around a cone, walk 3 metres back, and sit back in the original chair. This test is part of a test battery for evaluating mobility performance in older adults with knee OA^54^. Participants will be allowed to use the arm rests on the chair if desired. Instructions will guide the participant to complete the task as quickly as possible without compromising safety (i.e., no running or jogging). The researcher will start the stopwatch when the participant starts to rise from the chair and the time will be stopped when the participant is sitting back in the chair. Trials will be repeated twice and the fastest time will be recorded.

*Secondary Outcome Measures*

1. *Self-reported Outcomes*

- Centre for Epidemiological Studies Depression (CES-D) Scale
  - Depression will be assessed with the CES-D Scale, a 20-item scale developed for the general population^50^ with emphasis on affect. Elements of affect include mood, guilt, worthlessness, helplessness, appetite, and sleep. Data from the CES-D are valid and reliable in arthritic populations^50–52^. A score of ≥16 indicates that the person experienced depressive symptoms over the past week^50^ while scores of 7 have been reported in the general population^50^.
- Edmonton Frail Scale (EFS)
- Frailty will be assessed using the Edmonton Frail Scale (EFS). The EFS is a brief screening interview for older adults to assess frailty that is commonly used in both inpatient and outpatient settings. The scale covers 8 domains: cognition, general health status, functional independence, social support, medication use, nutrition, mood, continence, and functional performance (defined as performance on the Timed Up and Go [TUG] test)^53^. The test is scored out of 17, with higher scores indicating higher levels of frailty^53^. The EFS demonstrated moderate correlations with the Geriatrician’s Clinician Impression of Frailty (GCIF) index (r=0.64), medication (r=0.34) and age (r=0.27)^53^. Additionally, the EFS showed good construct validity with the Barthel Index (r=-0.58), and showed good inter-rater reliability with a Cronbach’s alpha of 0.62^53^.
- Co-Morbidity, Outside Events, and Feedback Form
  - At the end of the post-intervention visit at the MacMobilize Lab, participants will be asked about the development of any co-morbidities throughout the duration of the interventions. They will be asked about any injuries or outside events that occurred during the intervention that may affect the interpretation of their results. They will also be asked to bring and list any medications that they currently take for any of the co-morbidities listed.
  - Participants will also be asked to provide feedback (positives, negatives, and personal comments) on their experiences in the interventions. This will allow for participant input for development of future studies.

*B. Strength*

- Knee torque (isometric)
  - The peak torque developed during knee extension and flexion during a maximum voluntary isometric contraction will be measured by use of a Biodex System 2 isokinetic dynamometer. Data will be presented as Nm/kg. After a submaximal practice, 5 maximum-effort trials of isometric knee extension, with the knee joint positioned at 65°, will be recorded. The same protocol will be repeated for isometric knee flexion. The peak value for each of knee extension and flexion will be extracted. The reliability for these data are excellent (r=0.96)^59^.
- Grip strength
  - Low grip strength is linked to functional decrements in older adults^62^. Grip strength will be assessed using a Jamar hand dynamometer. The hand dynamometer will be set to a fixed position and all values will be expressed in both absolute (Nm) and relative (Nm/kg) values for comparison across participants. The protocol will involve the participants sitting in a chair with their elbow flexed 90 degrees. Gripping the dynamometer, the participant will be asked to squeeze the grip as hard as possible. Trials will be repeated three times for each the left and right hands. The peak value of each hand will be recorded and compared to normative data.

*C. Muscle and Fat Volume*

- Iterative Decomposition of water and fat with Echo Asymmetry and Least-squares estimation (IDEAL)
  - At the Imaging Research Center at St. Joseph’s Hospital in downtown Hamilton, thigh muscle and fat volumes will be calculated from scans acquired via 3.0T MRI images (3.0T MR750 Discovery research-grade scanner; GE Healthcare). The IDEAL sequence was chosen as it provides artifact-free images of fat fraction with great uniformity in comparison to chemical shift and opposed-phase imaging^63^.
  - For calibration, capsules of olive oil and saline will be placed on the skin for water-only and fat-only images. Three millimeter (mm) slices will be obtained from the thigh of the most symptomatic knee starting from 30% of the distance from the lesser trochanter to the tibiofemoral joint space using the IDEAL sequence: axial view, TE 31.512 msec, TR 2000 ms, matrix size 512x512, 60 slices, 140x110 mm field of view, slice gap 0, echo train length 6, bandwidth = 195.3 kHz, 1 NEX, and scan time = 7.25 minutes.

*D. Cartilage Morphology*

- Sodium (^23^Na) imaging will be obtained using the 3.0T MRI scanner at the Imaging Research Center.
  - Because of its high specificity for GAG content without a contrast agent, ^23^Na imaging is an excellent technique to detect cartilage degradation^64^. Sodium signal change correlated strongly (R^2^=0.85, p<0.001) with proteoglycan loss in knee cartilage *in vitro*^65^ and discriminated healthy from OA knees *in vivo*^66,67^.
  - For calibration, markers (capsule of 50, 100, 200 and 300 mmol/L ^23^Na concentration) will be placed on the skin. Prior to acquisition, we will acquire B_1_ maps, using a dual flip angle technique, and B_0_ maps using the ^1^H body coil (scan time=5 min). The ^23^Na images will be corrected for B_0_ and B_1_ inhomogeneity and the signal intensity of cartilage will be extracted in reference to calibration markers. Concentrations of ^23^Na are consistent in healthy cartilage; thus the mean and standard deviation of ^23^Na are useful measures of cartilage quality^64^.
  - Following ^1^H imaging, participants will be removed from the scanner and the coil will be switched for ^23^Na acquisition.  Following localization, ^23^Na images will be acquired using a 3D-CONES sequence^68^: FA 70**°,** TE=2.5 ms, TR=100 ms, 32 averages (scan time = 8.5 min) with a 2 mm isotropic resolution.

*E. Biomechanical Analyses of Gait and Static Postures*

- Participants will change into shorts, a sports bra, and be barefoot. The participants’ height, body mass, lower limb length, anterior superior iliac spine (ASIS) distance, posterior superior iliac spine (PSIS) distance, upper thigh circumference, abdominal circumference knee width, calf circumference and ankle width will be measured. Then, 3 rigid body marker clusters will be placed on the participant with Velcro straps. These will be placed between the left and right PSIS, on the study thigh, shank and the top of the foot. These correspond to the specifications required to create a lower-body model (C-Motion). Landmarks will be digitized in reference to the rigid body marker clusters. These landmarks include: both left and right anterior superior iliac spine, iliac crest, posterior superior iliac spine, greater trochanter, lateral and medial distal femoral condyles, lateral and medial tibial proximal condyles, lateral and medial malleoli, tibial tuberosity, proximal fibular head, head of metatarsals 1, 2, 5, and calcaneus.

With the participants on a force plate (AMTI) and in the field of view of three banks of Optotrak cameras (which record only positions of the markers), a 5 second standing reference trial will be collected for the motion capture system. Participants will be instructed to perform 10 hip rotations, 5 in each direction. Participants will be instructed to perform 5 knee flexions and extensions of both legs. Participants will be asked to perform 3 static postures (squat, lunge and wide-legged stance) for 10 seconds with at least a 10 second rest in between each posture. The order of these postures will be randomized between participants. Finally, participants will be asked to walk across the 10 foot motion capture area. For each yoga posture and gait, 3 successful trials will be recorded.

Protocol

*Baseline Measures:*

- *MacMobilize Laboratory at McMaster University, Hamilton*

1. Written, informed consent
2. Anthropometric Measurements
3. Resting Heart Rate and Blood Pressure
4. Instrumentation & Biomechanical Analyses (Gait and Yoga)
5. Mobility Performance Measures
6. Strength Assessment
7. Questionnaires: KOOS, ICOAP, CES-D, Frailty Index

This laboratory visit will require 2.5 hours to complete.

- *Imaging Research Center at St. Joseph’s Hospital (BE and TE groups only)*
  - Questionnaires and MRI eligibility
  - IDEAL sequence scan on thigh of most troublesome knee
  - Na^2+^ mapping of the cartilage of the most troublesome knee

This visit will require 1 hour to complete.

*Interventions:*

- *Biomechanical Exercise (BE); De La Sol Yoga at York and Locke Streets, Hamilton*
- 12-week intervention
- Requesting attendance to 3 supervised classes each week

Each class will be 1 hour.

- *Traditional Exercise (TE) Program; PACE at McMaster University, Hamilton*
  - 12-week intervention
  - Requesting attendance to 3 sessions each week

Each session is expected to last 1 hour.

- *Attention Control Meditation (M) Program; De La Sol Yoga at York and Locke Streets, Hamilton*
  - 12-week intervention
  - Requesting attendance to 3 supervised classes each week

Each class will be 1 hour.

*Follow-up Measures*

- *MacMobilize Laboratory at McMaster University, Hamilton*

1. Anthropometric Measurements
2. Resting Heart Rate and Blood Pressure
3. Instrumentation & Biomechanical Analyses (Gait and Yoga)
4. Mobility Performance Measures
5. Strength Assessment
6. Questionnaires: KOOS, ICOAP, CES-D, Frailty Index
7. Stipend

This laboratory visit will require 2.5 hours to complete.

- *Imaging Research Center at St. Joseph’s Hospital (BE and TE groups only)*
  - Questionnaires and MRI eligibility
  - IDEAL sequence scan on thigh of most troublesome knee
  - Na^2+^ mapping of the cartilage of the most troublesome knee

This visit will require 1 hour to complete.

Statistical Analyses

- Descriptive statistics will be determined for all of the outcome measures.
- Repeated measures analysis of variance will be used to determine if changes in the primary and secondary outcome measures as a result the interventions. Group by Time interactions will provide insight into which intervention provided the best tissue and clinical outcomes. Significance will be set to an alpha value of <0.05.
- Multiple linear regression analyses will be used to test the relationship of tissue outcomes with clinical outcomes. R-square and change in R-square will be evaluated to observe changes in explained variance.

**References**

1. Badley EM. *Arthritis and Related Conditions in Ontario: ICES Research Atlas*. Institute for Clinical Evaluative Sciences; 2004.

2. Guccione AA. Arthritis and the process of disablement. *Phys. Ther.* 1994;74(5):408-414.

3. Van Baar ME, Dekker J, Oostendorp RAB, Bijl D, Voorn TB, Bijlsma JWJ. Effectiveness of exercise in patients with osteoarthritis of hip or knee: nine months’ follow up. *Ann. Rheum. Dis.* 2001;60(12):1123–1130.

4. Zhang W, Moskowitz RW, Nuki G, et al. OARSI recommendations for the management of hip and knee osteoarthritis, Part I: Critical appraisal of existing treatment guidelines and systematic review of current research evidence. *Osteoarthritis Cartilage* 2007;15(9):981-1000.

5. Fransen M, McConnell S. Exercise for osteoarthritis of the knee. In: The Cochrane Collaboration, ed. *Cochrane Database of Systematic Reviews*. Chichester, UK: John Wiley & Sons, Ltd; 2008.

6. Robbins SM, Birmingham TB, Callaghan JP, Jones GR, Chesworth BM, Maly MR. Association of pain with frequency and magnitude of knee loading in knee osteoarthritis. *Arthritis Care Res.* 2011;63(7):991-997.

7. Kerrigan DC, Franz JR, Keenan GS, Dicharry J, Della Croce U, Wilder RP. The Effect of Running Shoes on Lower Extremity Joint Torques. *PM&R* 2009;1(12):1058-1063.

8. Miyazaki T, Wada M, Kawahara H, Sato M, Baba H, Shimada S. Dynamic load at baseline can predict radiographic disease progression in medial compartment knee osteoarthritis. *Ann. Rheum. Dis.* 2002;61(7):617–622.

9. Bennell KL, Bowles K-A, Wang Y, Cicuttini F, Davies-Tuck M, Hinman RS. Higher dynamic medial knee load predicts greater cartilage loss over 12 months in medial knee osteoarthritis. *Ann. Rheum. Dis.* 2011;70(10):1770-1774.

10. Bennell KL, Hunt MA, Wrigley TV, Lim B-W, Hinman RS. Role of Muscle in the Genesis and Management of Knee Osteoarthritis. *Rheum. Dis. Clin. N. Am.* 2008;34(3):731-754.

11. Sharma L, Dunlop DD, Cahue S, Song J, Hayes KW. Quadriceps strength and osteoarthritis progression in malaligned and lax knees. *Ann. Intern. Med.* 2003;138(8):613-619.

12. Kolasinski SL, Garfinkel M, Tsai AG, Matz W, Dyke AV, Schumacher Jr HR. Iyengar yoga for treating symptoms of osteoarthritis of the knees: a pilot study. *J. Altern. Complement. Med.* 2005;11(4):689–693.

13. Brenneman EC, Kuntz AB, Wiebenga EG, Maly MR. A strengthening program to minimize medial knee loads for knee OA: Effect on symptoms, mobility, strength, knee mechanics, and muscel activations. *Phys. Ther.* 2014;Submitted.

14. Longpré HS, Johnson AL, Maly MR. Identifying Yoga-Based Knee Strengthening Exercises with Minimal Medial Knee Loads. *Clin. Biomech.* 2014;Submitted.

15. Felson DT, Zhang Y, Hannan MT, et al. The incidence and natural history of knee osteoarthritis in the elderly, the framingham osteoarthritis study. *Arthritis Rheum.* 1995;38(10):1500–1505.

16. Felson DT, Zhang Y, Hannan MT, et al. Risk factors for incident radiographic knee osteoarthritis in the elderly. The Framingham Study. *Arthritis Rheum.* 1997;40(4):728–733.

17. Oliveria SA, Felson DT, Reed JI, Cirillo PA, Walker AM. Incidence of symptomatic hand, hip, and knee osteoarthritis among patients in a health maintenance organization. *Arthritis Rheum.* 1995;38(8):1134–1141.

18. Bennell KL, Wrigley TV, Hunt MA, Lim B-W, Hinman RS. Update on the role of muscle in the genesis and management of knee osteoarthritis. *Rheum. Dis. Clin. N. Am.* 2013;39(1):145-176.

19. Unruh AM. Gender variations in clinical pain experience. *Pain* 1996;65(2):123-167.

20. Felson DT, Naimark A, Anderson J, Kazis L, Castelli W, Meenan RF. The prevalence of knee osteoarthritis in the elderly. The Framingham Osteoarthritis Study. *Arthritis Rheum.* 1987;30(8):914–918.

21. Felson DT, Zhang Y. An update on the epidemiology of knee and hip osteoarthritis with a view to prevention. *Arthritis Rheum.* 1998;41(8):1343-1355.

22. Hochberg M, Kasper J, Williamson J, Skinner A, Fried L. The contribution of osteoarthritis to disability: preliminary data from the Women’s Health and Aging Study. *J. Rheumatol. Suppl.* 1995;43:16-18.

23. Altman R, Asch E, Bloch D, et al. Development of criteria for the classification and reporting of osteoarthritis: classification of osteoarthritis of the knee. *Arthritis Rheum.* 1986;29(8):1039–1049.

24. Kothari M, Guermazi A, von Ingersleben G, et al. Fixed-flexion radiography of the knee provides reproducible joint space width measurements in osteoarthritis. *Eur. Radiol.* 2004;14(9):1568-1573.

25. Binkley JM, Stratford PW, Lott SA, Riddle DL, others. The Lower Extremity Functional Scale (LEFS): scale development, measurement properties, and clinical application. *Phys. Ther.* 1999;79(4):371–383.

26. Hoogeboom TJ, de Bie RA, den Broeder AA, van den Ende CH. The Dutch Lower Extremity Functional Scale was highly reliable, valid and responsive in individuals with hip/knee osteoarthritis: a validation study. *BMC Musculoskelet. Disord.* 2012;13(1):117.

27. Pua Y-H, Cowan SM, Wrigley TV, Bennell KL. The Lower Extremity Functional Scale could be an alternative to the Western Ontario and McMaster Universities Osteoarthritis Index physical function scale. *J. Clin. Epidemiol.* 2009;62(10):1103-1111.

28. Williams VJ, Piva SR, Irrgang JJ, Crossley C, Fitzgerald GK. Comparison of Reliability and Responsiveness of Patient-Reported Clinical Outcome Measures in Knee Osteoarthritis Rehabilitation. *J. Orthop. Sports Phys. Ther.* 2012;42(8):716-723.

29. Yeung TSM, Wessel J, Stratford P, MacDermid J. Reliability, Validity, and Responsiveness of the Lower Extremity Functional Scale for Inpatients of an Orthopaedic Rehabilitation Ward. *J. Orthop. Sports Phys. Ther.* 2009;39(6):468-477.

30. Bolton JE, Wilkinson RC. Responsiveness of pain scales: a comparison of three pain intensity measures in chiropractic patients. *J. Manipulative Physiol. Ther.* 1998;21(1):1-7.

31. Stratford PW, Spadoni G. The reliability, consistency, and clinical application of a numeric pain rating scale. *Physiother. Can.* 2001;53(2):88-91.

32. Ferraz MB, Quaresma M, Aquino L, Atra E, Tugwell P, Goldsmith C. Reliability of pain scales in the assessment of literate and illiterate patients with rheumatoid arthritis. *J. Rheumatol.* 1990;17(8):1022-1024.

33. Jensen MP, Karoly P, Braver S. The measurement of clinical pain intensity: a comparison of six methods. *Pain* 1986;27(1):117-126.

34. Jensen MP, Turner JA, Romano JM, Fisher LD. Comparative reliability and validity of chronic pain intensity measures. *Pain* 1999;83(2):157–162.

35. Berthier F, Potel G, Leconte P, Touze M-D, Baron D. Comparative study of methods of measuring acute pain intensity in an ED. *Am. J. Emerg. Med.* 1998;16(2):132-136.

36. Paradowski PT, Bergman S, Sundén-Lundius A, Lohmander LS, Roos EM. Knee complaints vary with age and gender in the adult population. Population-based reference data for the Knee injury and Osteoarthritis Outcome Score (KOOS). *BMC Musculoskelet. Disord.* 2006;7(1):38.

37. Roos EM, Klässbo M, Lohmander LS. WOMAC Osteoarthritis Index: Reliability, validity, and responsiveness in patients with arthroscopically assessed osteoarthritis. *Scand. J. Rheumatol.* 1999;28(4):210–215.

38. Frobell RB, Svensson E, Göthrick M, Roos EM. Self-reported activity level and knee function in amateur football players: the influence of age, gender, history of knee injury and level of competition. *Knee Surg. Sports Traumatol. Arthrosc.* 2008;16(7):713-719.

39. Ageberg E, Forssblad M, Herbertsson P, Roos EM. Sex Differences in Patient-Reported Outcomes After Anterior Cruciate Ligament Reconstruction: Data From the Swedish Knee Ligament Register. *Am. J. Sports Med.* 2010;38(7):1334-1342.

40. Alviar M, Olver J, Brand C, Hale T, Khan F. Do patient-reported outcome measures used in assessing outcomes in rehabilitation after hip and knee arthroplasty capture issues relevant to patients? Results of a systematic review and ICF linking process. *J. Rehabil. Med.* 2011;43(5):374-381.

41. Collins NJ, Misra D, Felson DT, Crossley KM, Roos EM. Measures of knee function: International Knee Documentation Committee (IKDC) Subjective Knee Evaluation Form, Knee Injury and Osteoarthritis Outcome Score (KOOS), Knee Injury and Osteoarthritis Outcome Score Physical Function Short Form (KOOS-PS), Knee Ou. *Arthritis Care Res.* 2011;63(S11):S208-S228.

42. Collins NJ, Roos EM. Patient-reported outcomes for total hip and knee arthroplasty: commonly used instruments and attributes of a “good” measure. *Clin. Geriatr. Med.* 2012;28(3):367-394.

43. Hawker GA, Davis AM, French MR, et al. Development and preliminary psychometric testing of a new OA pain measure – an OARSI/OMERACT initiative. *Osteoarthritis Cartilage* 2008;16(4):409-414.

44. Hawker GA, Stewart L, French MR, et al. Understanding the pain experience in hip and knee osteoarthritis – an OARSI/OMERACT initiative. *Osteoarthritis Cartilage* 2008;16(4):415-422.

45. Davis AM, Lohmander LS, Wong R, Venkataramanan V, Hawker GA. Evaluating the responsiveness of the ICOAP following hip or knee replacement. *Osteoarthritis Cartilage* 2010;18(8):1043-1045.

46. Lorig K, Chastain RL, Ung E, Shoor S, Holman HR. Development and evaluation of a scale to measure perceived self-efficacy in people with arthritis. *Arthritis Rheum.* 1989;32(1):37–44.

47. Brady TJ. Measures of self-efficacy: Arthritis Self-Efficacy Scale (ASES), Arthritis Self-Efficacy Scale-8 Item (ASES-8), Children’s Arthritis Self-Efficacy Scale (CASE), Chronic Disease Self-Efficacy Scale (CDSES), Parent’s Arthritis Self-Efficacy Scale (PASE), an. *Arthritis Care Res.* 2011;63(S11):S473-S485.

48. Keefe FJ, Lefebvre JC, Maixner W, Salley AN, Caldwell DS. Self-efficacy for arthritis pain: Relationship to perception of thermal laboratory pain stimuli. *Arthritis Rheum.* 1997;10(3):177–184.

49. Buescher K, Johnston J, Parker J, et al. Relationship of self-efficacy to pain behavior. *J. Rheumatol.* 1991;18(7):968-972.

50. Radloff LS. The CES-D scale a self-report depression scale for research in the general population. *Appl. Psychol. Meas.* 1977;1(3):385-401.

51. Blalock SJ, Devellis RF, Brown GK, Wallston KA. Validity of the Center for Epidemiological Studies Depression Scale in arthritis populations. *Arthritis Rheum.* 1989;32(8):991–997.

52. Weissman MM, Sholomskas D, Pottenger M, Prusoff BA, Locke BZ. Assessing depressive symptoms in five psychiatric populations: a validation study. *Am. J. Epidemiol.* 1977;106(3):203–214.

53. Rolfson DB, Majumdar SR, Tsuyuki RT, Tahir A, Rockwood K. Validity and reliability of the Edmonton Frail Scale. *Age Ageing* 2006;35(5):526-529.

54. Dobson F, Hinman RS, Roos EM, et al. OARSI recommended performance-based tests to assess physical function in people diagnosed with hip or knee osteoarthritis. *Osteoarthritis Cartilage* 2013;21(8):1042-1052.

55. ATS Committee on Proficiency Standards for Clinical Pulmonary Function Laboratories. ATS statement: guidelines for the six-minute walk test. *Am. J. Respir. Crit. Care Med.* 2002;166(1):111.

56. Cahalin LP. The Six-Minute Walk Test Predicts Peak Oxygen Uptake and Survival in Patients With Advanced Heart Failure. *CHEST J.* 1996;110(2):325.

57. McCarthy EK, Horvat MA, Holtsberg PA, Wisenbaker JM. Repeated chair stands as a measure of lower limb strength in sexagenarian women. *J. Gerontol. A. Biol. Sci. Med. Sci.* 2004;59(11):1207–1212.

58. Smith WN, Del Rossi G, Adams JB, et al. Simple equations to predict concentric lower-body muscle power in older adults using the 30-second chair-rise test: a pilot study. *Clin. Interv. Aging* 2010;5:173.

59. Gross MT, Huffman GM, Phillips CN, Wray JA. Intramachine and intermachine reliability of the Biodex and Cybex® II for knee flexion and extension peak torque and angular work. *J. Orthop. Sports Phys. Ther.* 1991;13(6):329-335.

60. Bean JF, Kiely DK, Herman S, et al. The relationship between leg power and physical performance in mobility‐limited older people. *J. Am. Geriatr. Soc.* 2002;50(3):461-467.

61. Bassey EJ, Fiatarone MA, O’neill EF, Kelly M, Evans WJ, Lipsitz LA. Leg extensor power and functional performance in very old men and women. *Clin. Sci.* 1992;82(3):321-327.

62. Rantanen T, Guralnik JM, Foley D, et al. Midlife hand grip strength as a predictor of old age disability. *Jama* 1999;281(6):558-560.

63. Bernard CP, Liney GP, Manton DJ, Turnbull LW, Langton CM. Comparison of fat quantification methods: A phantom study at 3.0T. *J. Magn. Reson. Imaging* 2008;27(1):192-197.

64. Choi J-A, Gold GE. MR Imaging of Articular Cartilage Physiology. *Magn. Reson. Imaging Clin. N. Am.* 2011;19(2):249-282.

65. Borthakur A. Sensitivity of MRI to proteoglycan depletion in cartilage: comparison of sodium and proton MRI. *Osteoarthritis Cartilage* 2000;8(4):288-293.

66. Hani AFM, Kumar D, Malik AS, Razak R. Physiological assessment of in vivo human knee articular cartilage using sodium MR imaging at 1.5T. *Magn. Reson. Imaging* 2013;31(7):1059-1067.

67. Newbould RD, Miller SR, Upadhyay N, et al. T1-Weighted Sodium MRI of the Articulator Cartilage in Osteoarthritis: A Cross Sectional and Longitudinal Study. Yang X, ed. *PLoS ONE* 2013;8(8):e73067.

68. Gurney PT, Hargreaves BA, Nishimura DG. Design and analysis of a practical 3D cones trajectory. *Magn. Reson. Med.* 2006;55(3):575-582.
